# Pharmacological consequences of the coexpression of BK channel α and auxiliary β subunits

**DOI:** 10.3389/fphys.2014.00383

**Published:** 2014-10-10

**Authors:** Yolima P. Torres, Sara T. Granados, Ramón Latorre

**Affiliations:** ^1^Departamento de Nutrición y Bioquímica, Facultad de Ciencias, Pontificia Universidad JaverianaBogotá, Colombia; ^2^Facultad de Ciencias, Centro Interdisciplinario de Neurociencia de Valparaíso, Universidad de ValparaísoValparaíso, Chile

**Keywords:** BK channel, *Slo1*, KCNMB, BK β subunits, BK pharmacology, auxiliary subunits

## Abstract

Coded by a single gene (*Slo1*, KCM) and activated by depolarizing potentials and by a rise in intracellular Ca^2+^ concentration, the large conductance voltage- and Ca^2+^-activated K^+^ channel (BK) is unique among the superfamily of K^+^ channels. BK channels are tetramers characterized by a pore-forming α subunit containing seven transmembrane segments (instead of the six found in voltage-dependent K^+^ channels) and a large C terminus composed of two regulators of K^+^ conductance domains (RCK domains), where the Ca^2+^-binding sites reside. BK channels can be associated with accessory β subunits and, although different BK modulatory mechanisms have been described, greater interest has recently been placed on the role that the β subunits may play in the modulation of BK channel gating due to its physiological importance. Four β subunits have currently been identified (i.e., β1, β2, β3, and β4) and despite the fact that they all share the same topology, it has been shown that every β subunit has a specific tissue distribution and that they modify channel kinetics as well as their pharmacological properties and the apparent Ca^2+^ sensitivity of the α subunit in different ways. Additionally, different studies have shown that natural, endogenous, and synthetic compounds can modulate BK channels through β subunits. Considering the importance of these channels in different pathological conditions, such as hypertension and neurological disorders, this review focuses on the mechanisms by which these compounds modulate the biophysical properties of BK channels through the regulation of β subunits, as well as their potential therapeutic uses for diseases such as those mentioned above.

## Introduction

**Figure 1:**
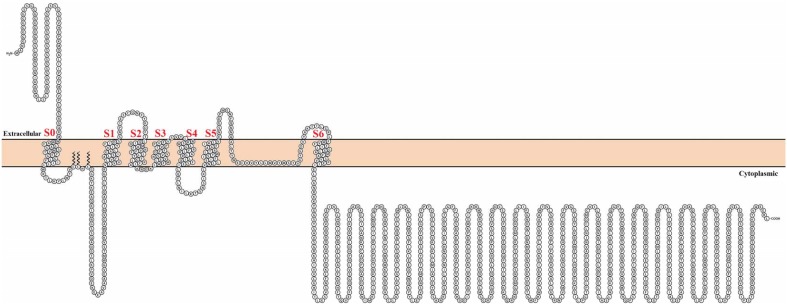
**Topological representation of the human Calcium-activated potassium channel α subunit**. Using Protter visualizator (Omasits et al., 2014), UniProt protein accession: Q12791.

Consisting of 5 families and more than 70 different encoding genes in mammals, the diversity of K^+^ channels is amazingly large (for a comprehensive review on K^+^ channels, see González et al., 2012). In particular, the Ca^2+^- and voltage-activated K^+^ (BK) channel is a relative of the 6-transmembrane domain voltage-dependent K^+^ (Kv) channel family, which is also part of the S4 superfamily encompassing voltage-dependent Na^+^ and Ca^2+^ channels. There are, however, several differences between Kv and BK channels that make BK channels unique. First, BK channels are encoded by a single gene (*Slo1*). Second, they contain seven transmembrane domains and, hence, the N-terminus is in contact with the cell external milieu (Meera et al., 1997) (Figure 1). Third, the BK channel can be independently activated by Ca^2+^ or voltage, and it can open in the absence of Ca^2+^. Moreover, it is clear at present that voltage and Ca^2+^ sensors are allosterically coupled to channel opening (as reviewed in Latorre et al., 2010; Horrigan, 2012). In other words, voltage or Ca^2+^ alone can open the channel, but the free energy required to open the channel greatly decreases when both sensors are activated. Finally, unlike Kv channels, where most voltage-sensing charges are contained in the S4 transmembrane segment, only about 50% of the gating charges for BK channels are in S4 and the rest of the voltage-sensing particles reside in S3 and S2 (Ma et al., 2006). Accordingly, the BK voltage sensor can be defined as a decentralized voltage sensor.

The BK channel in mammals is ubiquitously distributed in different tissues and, because it is activated by voltage and Ca^2+^, it is the perfect molecular machine to reduce or stop excitatory stimuli. For example, the BK channel modulates neurotransmitter release by co-localizing with voltage-dependent Ca^2+^ channels (Robitaille and Charlton, 1992). In vascular smooth muscle cells, BK channels regulate contractile tone. In this case, increments in local Ca^2+^ (i.e., Ca^2+^ sparks) produce BK channel-mediated spontaneous transient outward currents (STOCs), thus hyperpolarizing the membrane and producing muscle relaxation (Jaggar et al., 2000; Ledoux et al., 2006). On the other hand, alterations in BK channels are known to be important in the pathophysiology of hypertension, asthma, diabetes, and epilepsy (as reviewed in Contreras et al., 2013). Because of the profound involvement of BK channels in the health problems described above, their activators, and blockers have potential therapeutic implications. In several cases, the action of these compounds is mediated by their binding to the β subunit or modulated by the presence of these subunits. Hence, the main goal of the present review is to provide an overview of our current knowledge of how such mediation and modulation are accomplished.

## BK channel β subunits

**Figure 2:**
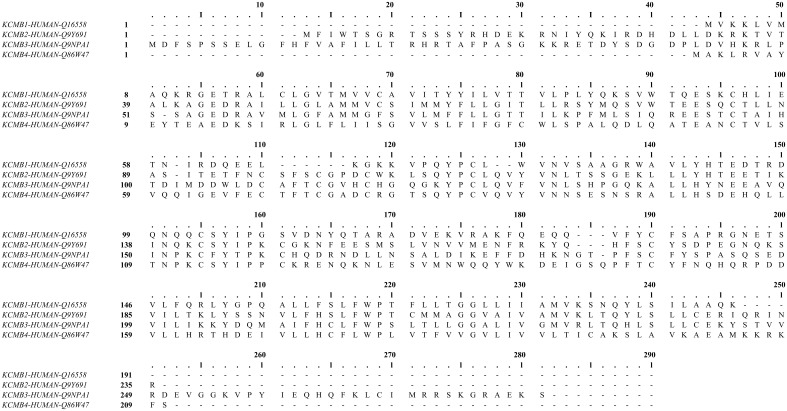
**Human calcium-activated potassium channel β subunit alignment**. Using MUSCLE software (Edgar, 2004). UniProt protein accession: KCMB1, Q16558; KCMB2, Q9Y691; KCMB3, Q9NPA1; KCMB4, Q86W47.

**Figure 3:**
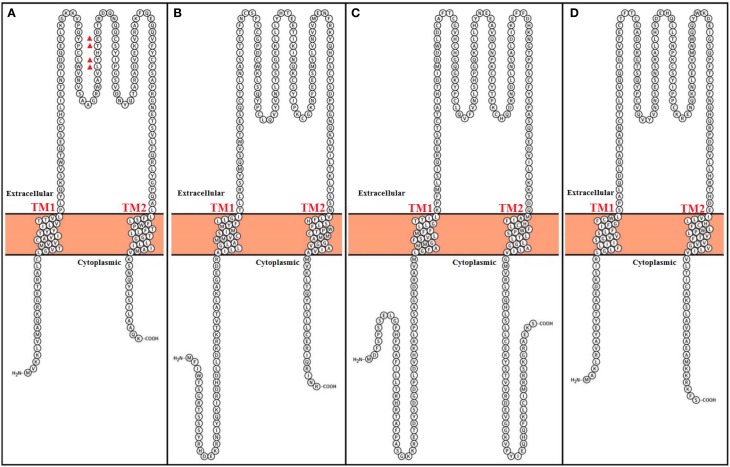
**Topological representation of human Calcium-activated potassium channel β subunits**. Using Protter visualizator (Omasits et al., 2014). UniProt protein accession: **(A)** KCMB1: Q16558, **(B)** KCMB2: Q9Y691, **(C)** KCMB3: Q9NPA1, **(D)** KCMB4: Q86W47.

In most tissues, the BK channel is associated with accessory β subunits. To date, four β subunits (β1–β4, encoded by the genes KCNMB1–4) have been identified (Figure 2) and their expression depends on cell type (as reviewed in Contreras et al., 2013) (Table 1). All β subunits share the same predicted structural characteristics (170–200 residues in length) and they are composed of two transmembrane segments (i.e., TM1 and TM2), intracellular N and C termini and a large extracellular loop (as reviewed in Orio et al., 2002; Torres et al., 2007; Hoshi et al., 2013b). The first β subunit to be identified was β1 in tracheal smooth muscle cells (Figure 3A) (Garcia-Calvo et al., 1994; Knaus et al., 1994). In heterologous systems such as *Xenopus oocytes* and cell cultures, the co-expression of β1 with the α subunit leads to an increase in the apparent Ca^2+^ sensitivity of the channel, which slows down activation and deactivation kinetics and causes a lefward shift in G-V relations by −70 to −80 mV at 5–10 μM intracellular Ca^2+^ concentrations (McManus et al., 1995; Wallner et al., 1995; Meera et al., 1996; Tanaka et al., 1997; Brenner et al., 2000a; Cox and Aldrich, 2000; Nimigean and Maglebly, 2000; Bao and Cox, 2005; Orio and Latorre, 2005).

**Table 1:** **Tissue expression of the BK channel accessory β subunits**.

**Subunit**	**Chromosome location (human)**	**Gene symbol (human)**	**UniProt protein accession number (human)**	**Tissue expression**
β1	5q34	KCNMB1	Q16558	Smooth muscle (Knaus et al., 1994), kidney, urinary bladder (Wu and Marx, 2010), brain (Tseng-Crank et al., 1996), myometrium (Wallner et al., 1996)
β2	3q26.32	KCNMB2	Q9Y691	Pancreas (Ohya et al., 2010), kidney, (Uebele et al., 2000), chromafin cells (Xia et al., 1999), brain (Wallner et al., 1999)
β3a	3q26.3-q27	KCNMB3	Q9NPA1-2	Spleen, placenta, pancreas, heart, kidney (Behrens et al., 2000; Brenner et al., 2000b; Uebele et al., 2000; Xia et al., 2000)
β3b	3q26.3-q27	KCNMB3	Q9NPA1-4	Lung, liver, testes, spleen, placenta, pancreas, heart, kidney, brain (Behrens et al., 2000; Brenner et al., 2000b; Xia et al., 2000)
β3c	3q26.3-q27	KCNMB3	Q9NPA1-3	Ovary, brain, lung, liver, spleen, placenta, pancreas, prostate, kidney (Behrens et al., 2000; Brenner et al., 2000b; Xia et al., 2000)
β3d	3q26.3-q27	KCNMB3	Q9NPA1-1	Lung, brain, testes, spleen, placenta, pancreas, kidney (Behrens et al., 2000; Brenner et al., 2000b; Xia et al., 2000)
β4	12q	KCNMB4	Q86W47	Brain, neuronal tissue, Behrens et al., 2000; Brenner et al., 2000b kidney, bladder smooth muscle (Chen and Petkov, 2009)

Functional coupling between α and β1 subunits is determined by the S0 transmembrane segment of the BK channel. Cysteine crosslinking experiments have indicated that β1 lies between and can interact with the voltage sensors of two adjacent α subunits, and that TM2 lies in the proximity of S0 (Wallner et al., 1996; Liu et al., 2010). Similar to the β1 subunit, β2 also increases apparent BK channel Ca^2+^ sensitivity and alters its gating kinetics, but the existence of 31 more amino acid residues in the N terminal has shown to promote an inactivation process (Figure 3B) (Wallner et al., 1999; Brenner et al., 2000a; Xia et al., 2003; Orio and Latorre, 2005). Although it behaves as an open channel block (Wallner et al., 1999), this N-type inactivation does not promote charge inmobilization, as in the N-type inactivation observed in Shaker K^+^ channels (Savalli et al., 2007). It is important to mention that although the β1 subunit can exist in a 1:1 stoichiometry with α subunits, there is a report suggesting that the variability seen in BK inactivation behavior in rat chromaffin cells can originate from a less than a 1:1 assembly of β2 and α subunits (Knaus et al., 1994; Ding et al., 1998; Wang et al., 2002).

The β3 subunit is less similar to the β1 and β2 subunits (Figure 3C) and confers distinct modulating characteristics to the α subunit (Riazi et al., 1999; Xia et al., 2000). There are four different β3 subunits (β3a–d), which all originate from alternative splicing of the same gene (i.e., KCNMB3) (Brenner et al., 2000b; Uebele et al., 2000). It was reported that TM1 is closest to αS1 and S2 and TM2 is closest to αS0 (Wu et al., 2013). The expression of β3a, b, and c causes a partial inactivation of potassium currents. Althoug β3a and c induce a similar inactivation process as the β2 subunit, β3b-dependent inactivation is faster. The β3d subunit does not causes inactivation of potassium currents (Brenner et al., 2000b; Uebele et al., 2000).

Similar to β1, TM1 from β4 subunit is nearest to αS1 and S2 meanwhile β4 TM2 is closest to S0 (Wu et al., 2009). This subunit its expressed in the brain and is considered to be a downregulator of BK channels by dramatically decelerating BK channel activation gating kinetics (Figure 3D) (Weiger et al., 2000). However, although β4 shifts conductance-voltage curves to the right along the voltage axis at Ca^2+^ concentrations lower that 1 μM, it increases apparent Ca^2+^ sensitivity at Ca^2+^ concentrations larger than 1 μM (Brenner et al., 2000b; Ha et al., 2004; Wang et al., 2006). Additionally, the β4 subunit reduces the voltage dependence of the conductance–voltage relationship as well as the slope of the gating charge-voltage curve (Wang et al., 2006; Contreras et al., 2012).

## The role of β subunits in BK channel pharmacology

BK channels can be modulated by diverse molecules that may induce either an increase or decrease in channel activity. The effects of these molecules can be exerted through direct interactions with the α subunit and, in some cases, by the expression of β subunits, which would modify such effects. Nevertheless, some of these molecules need β subunits to modulate BK channel activity. The effects of various molecules that have been extensively studied as BK channel modulators and whose activity has been proposed to be related to β subunit expression will be described here (Table 2). Among the latter, toxins (such as charybdotoxin, iberiotoxin), ethanol, steroids, and fatty acids (like araquidonic acid and metabolites) are also mentioned. Molecules like tungstate, DiBAC_4_(bis (1,3-dibutylbarbituric acid) trimethine oxonol), nitric oxide (NO) and tetrandrine, whose effects are determined by β subunits and have not been studied in detail will be also described.

**Table 2:** **Molecules that regulate BK channel activity through β subunits**.

	**Ligand**	**Subunit associated to a modulatory effect on the BK channel activity**
Toxins	Charybdotoxin	β1, β2, β3, and β4
	Iberiotoxin	β1 and β4
	Slotoxin	β1 and β4
	Martentoxin	β4
	Vt 3.1	β4
Fatty acids	Arachidonic acid	β1, β2, and β3
	Docosahexaenoic	β1 and β4
	Eicosapentaenoic	β1
	Acil-CoA	β2
Alcohol	Ethanol	β1 and β4
Steroids	17β-Estradiol	β1
	Tamoxifen	β1
	Dehydroepiandrosterone	β2 and β4
	Lithocolic acid	β1
	Dehydrosoyasaponin-I	β1
Others	Tungstate	β1
	DiBAC_4_	β1, β2, and β4

### Toxin interaction

**Figure 4:**
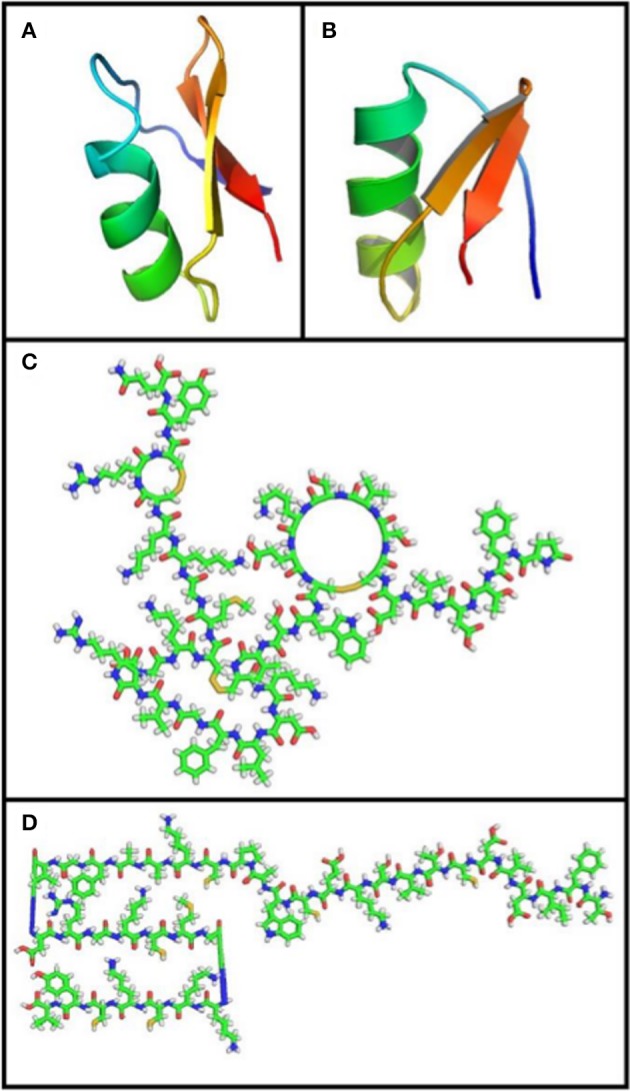
**Toxin structure**. **(A)** Charybdotoxin: PDB ID 2CRD; (Bontems et al., 1992). **(B)** Martentoxin: PDB ID 1M2S (Wang et al., 2005). **(C)** Iberiotoxin: PubChem ID 16132435. **(D)** Slotoxin: PubChem ID 16133816. Using PyMOL Molecular Graphics System, Version 1.5.0.4 Schrödinger, LL. Green: carbon atoms, white: hydrogen atoms, red: oxygen atoms, blue: nitrogen atoms, yellow: sulfur atoms.

BK channels have been described to be sensitive to scorpion-peptide toxins, such as charybdotoxin (ChTX), iberiotoxin (IbTX), slotoxin (SloTX), and martentoxin (Figure 4). Toxins can affect channel properties with different affinities and specificities, and their binding has been shown to be modulated by the coexpression of β subunits. For example, while IbTX—which is a 37 amino acid peptide present in the venom of the African scorpion *Buthus tamulus* (Galvez et al., 1990)—is a specific BK channel blocker (Kaczorowski and Garcia, 1999), ChTX—which is a peptide with the same number of amino acid residues isolated from the scorpion *Liurus quinquestriatus*—blocks other potassium channels (Galvez et al., 1990; Kaczorowski and Garcia, 1999). Interestingly, both toxins reversibly block BK channels by externally binding to the mouth of the channel through a bimolecular reaction (Miller et al., 1985; Anderson et al., 1988; Candia et al., 1992; Giangiacomo et al., 1992). IbTX and ChTX blockade is characterized by long-lived (blocked) non-conducting states that separate bursts of activity. These long-lived blocked states define mean blocked times of 10 and 200 s for ChTX and IbTX, respectively. Under similar experimental conditions, the dissociation constant (Kd ~1.5 nM) for IbTX is nearly 10-fold smaller than for ChTX (Candia et al., 1992; Giangiacomo et al., 1992; Gribkoff et al., 1996; Mullmann et al., 1999).

**Figure 5:**
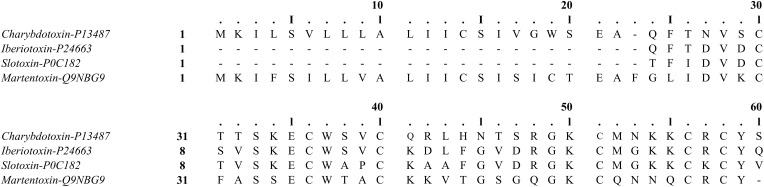
**Sequence alignment of the ChTX family of K^+^ toxins**. Using MUSCLE software (Edgar, 2004). UniProt protein accession: Charybdotoxin, P13487; Iberiotoxin, P24663; Slotoxin, P0C182; Martentoxin, Q9NBG9.

It has been shown that ChTX and IbTX toxins have different net charges that may affect selectivity by means of a mechanism involving electrostatic interactions with BK channels. Since the channel external vestibule has a fixed negative charge density region, a local electrostatic potential is set up near the toxin binding site, raising the local concentration of the positively-charged toxin (Anderson et al., 1988). Although ChTX and IbTX are equal in size and display a highly homologous sequence (68% homologous), ChTX has a net charge of +5 due to the presence of several basic residues, while the net charge for IbTX is +2, since it has 4 more acidic residues and 1 basic amino acid residue less than the ChTX (Figure 5). The latter would explain the different behaviors observed in their toxin-channel electrostatic interactions where the binding of ChTX to BK channel is more sensitive to ion strength variations that IbTX binding (Galvez et al., 1990; Candia et al., 1992; Giangiacomo et al., 1992). Additionally, it has been suggested that an electrostatic mechanism underlies the dissociation of ChTX from the channel (Anderson et al., 1988; MacKinnon and Miller, 1988). The apparent voltage dependence of the channel block is due to a destabilization of the channel-toxin complex, which is mediated by K^+^ ions entering the selectivity filter from the internal side when the membrane is depolarized, hence inducing a repulsion force that unbounds the toxin from the channel (MacKinnon and Miller, 1988).

These two toxins plug the BK channel by means of direct interaction between toxin lysine 27 and the channel selectivity filter (Park and Miller, 1992; Mullmann et al., 1999). The above studies led to the hypothesis that lysine 27 seems to be in the proximity of one of the external K^+^ binding sites. This hypothesis has recently been fully confirmed by Banerjee et al. (2013), who were able to solve the crystal structure of the chimeric channel Kv1.2/Kv2.1 in complex with ChTX. The structure of the Kv-ChTX complex shows that the toxin binds to the extracellular aspect of the selectivity filter and the most extracellular K^+^ binding site is devoid of K^+^ ions.

Coexpression of BK channel α and β subunits causes a dramatic effect on ChTX-BK interaction properties when compared to the α subunit alone. It has been suggested that extracellular loop from β subunit is involved in the formation of the toxin receptor (Hanner et al., 1997). The affinity of ChTX to α +β1 channels is 50-fold larger than that of channels formed by the α subunit alone. This increase in toxin binding potency is due to a seven-fold decrease in the toxin dissociation rate constant and an increase of about five-fold in the association rate constant (Hanner et al., 1997). To identify the β1 subunit amino acid residues involved in ChTX binding affinity changes, an alanine scanning mutagenesis along the β1 external loop was performed. The results indicated that the L90, Y91, W93, and E94 residues are critical for ChTX high-affinity binding to the α + β1 BK channel. Mutations to alanine of these residues do not change the physical interactions between β1 and α subunits. However, they affect ChTX association and dissociation kinetics, suggesting that the mutated residues may be significantly involved in the large increases observed in ChTX binding affinity in the presence of the β1 subunit (Hanner et al., 1998).

It is worthy of noting that studies have shown that BK channels expressing auxiliary subunits other than β1 change their affinity to toxins in an opposite way, that is, by inducing a decrease in the toxin association rate. For instance, Reinhart et al. (1989) describe channels with two different sensitivities to ChTX in brain neurons, namely that while one type of BK channel was blocked with concentrations similar to those required to block the BK channel from skeletal muscle membranes, as describe by Anderson et al. (1988), the others were rather insensitive to the toxin. Further studies on rat supraoptic magnocellular neurons reported by Dopico et al. (1999b) showed similar results, where some BK channels were blocked at ChTX or IbTX concentrations of 10 nM, while the others were insensitive to ChTX even at concentrations as large as 360 nM. To explain such differences in sensitivity, Meera et al. (2000) reported that coexpression of α with the β4 subunit (which is the β subunit present in the brain) leads to a BK channel phenotype that displays very low ChTX and IbTX sensitivity. Meera et al. (2000) proposed that the molecular basis for toxin insensitivity in BK channels found in neurons is that they are formed by the α + β4 subunit combination (Behrens et al., 2000; Meera et al., 2000). β4 subunit expression induces a rise in the apparent half-maximal inhibitory concentration (IC_50_) of the BK channel and a lessening of the toxin association rate by 250–1000-fold. To explain the mechanism that may determine the differences in effects induced by the β1 and β4 subunits on ChTX binding to BK channels, the smooth muscle β1 subunit extracellular loop was exchanged with the neuronal β4 subunit loop. The results showed that the β subunit extracellular loop determined the affinity of this toxin to the BK channel. The β4 subunit external loop contains more basic residues (e.g., K120, R121, K125) than the β1 subunits, and their neutralization, or charge reversals (i.e., R121D) greatly increase toxin association and dissociation rates. These results suggest that positively charged residues impose an electrostatic shield on ChTX action (Gan et al., 2008). When comparing β1 and β4 subunits, it has been shown that β4 is missing a conserved Y100 residue, and restoring this residue into the β4 subunit enables the recovery of the α + β4 BK channel's higher sensitivity to ChTX. In addition, tyrosine 294 at the C-terminal end of the pore loop in *mslo*, which interacts with Y36 in ChTX, as well as the missing β4 Y100 residue are possible sites for ChTX channel interaction (Gao and Garcia, 2003; Gan et al., 2008).

These results have also been confirmed by studies evaluating IbTX affinity to BK channels, where the presence of β4 leads to reduced toxin binding to the channels. However, unlike ChTX, the presence of β1 decreases IbTX association rates by about 40-fold and produces a large decrease (~100-fold) in dissociation rates. The end result of this is that although α + β1 BK channels show less sensitivity to IbTX, binding is essentially irreversible (Meera et al., 2000). These results strongly suggest that differences in toxin residues determine combinatorial effects with the extracellular loop of the β subunit. However, it is important to consider that some reports have found that IbTX behaves similar to ChTX in the presence of β1. This discrepancy can be due to differences in the ionic strengths of the solutions used to perform the experiments (Hanner et al., 1997; Meera et al., 2000).

Similar to the effects observed in channels expressing α + β4 subunits, the expression of β2 was found to trigger a steeper decrease in response rates to ChTX compared to the effect on the α subunit alone [EC_50_ = 58 nM and 1 nM, respectively] (Wallner et al., 1999). This has been further corroborated in chromaffin cells, where the β2 subunit is endogenously expressed (Ding et al., 1998). Additionally, studies evaluating the coexpression of α + β3 channels also report a decrease in the degrees of blockade by ChTX (IC_50_ 80 nM) (Xia et al., 1999, 2000; Zeng et al., 2008), together with a change in the magnitude and kinetics of the blocking reaction (Ding et al., 1998). Different to what has been found when comparing amino acid sequences of β1 and β4, residues proposed to mediate ChTX interaction with β1 are present in the β3 subunit, which suggests that other differences between both subunits are responsible for dissimilarities in ChTX sensitivity (Xia et al., 1999).

Although the previous results strongly suggest that amino acid sequences in the β subunit and toxin loop play an important role in toxin affinity to the BK channel, IbTX, and ChTX surface charge interfaces have been found to be nearly identical. This means that peptide net charge does not contribute to their potassium channel specificities, suggesting that the molecular mechanism responsible for such effects would be related to glycosylations in the β subunit extracellular loop and its proximity to the channel vestibule (Gao and Garcia, 2003).

Another scorpion toxin from the *Centruroides noxius* species that specifically inhibits mammalian BK channels is Slotoxin (SloTX), which has 76% of identity to IbTX and 54% to ChTX (Garcia-Valdes et al., 2001). Like IbTX and ChTX, SloTX interacts through a bimolecular reaction by blocking BK α subunit pore domain. In addition, the toxin can differentiate between the channels expressing α, α + β1, and α + β4 subunits. If the β1 subunit is coexpressed with the α subunit, on-rates of channel blockade are reduced by two orders of magnitude compared to when the BK channel α subunit is alone. Furthermore, off-rates acutely decrease, making SloTX blockade irreversible. Toxin association rates in oocytes coexpressing α + β4 subunits are much slower than when β1 is present. Therefore, α + β4 channels are extremely resistant to SloTX blockade, but once blocked, toxin interaction seems irreversible (Garcia-Valdes et al., 2001).

Martentoxin, which is a peptide isolated from *Buthus martensi Karsch* scorpion venom, can bind to K^+^ channels by means of a similar mechanism as other well-known scorpion toxins (Ji et al., 2003; reviewed in Tao et al., 2012). Martentoxin preferentially selects BK over Kv channels with a 1000-fold difference (Shi et al., 2008). After blocking BK channel currents with martentoxin, full recovery is achieved much faster than with ChTX (Ji et al., 2003). Martentoxin is a unique neurotoxin whose activity depends on Ca^2+^ concentrations, and which selectively blocks α + β4 subunit BK channels with a higher preference than channels expressing the α subunit alone (Shi et al., 2008; Tao et al., 2014). At low Ca^2+^ cytoplasmic concentrations, neuronal α + β4 BK channel currents are inhibited by martentoxin, but at high Ca^2+^ concentrations the toxin acts as an agonist. Martentoxin does not shift BK channel (α + β1) conductance-voltage dependence, thus indicating that this toxin does not interact with the voltage sensor, unlike β1 (Tao et al., 2011). After applying 400 nM of martentoxin, the current induced by αBK channels by a +80 mV pulse was not significantly affected, despite the fact that 400 nM of IbTX abolishes it completely (Shi et al., 2008). This result suggests that BK channels expressing only α subunits are insensitive to martentoxin, which could make it a potential selector for BK channel subtypes (Tao et al., 2011).

The toxin Vt3.1 is a disulfide-cross-linked dimer conopeptide isolated from *Conus vitulinus* that acts on BK channels activity by a different mechanism of that reported for toxins like ChTX and IbTX. Toxin Vt3.1 inhibits α BK channels. However, its effect is substantially enhanced when channels are formed by α + β4. This property makes the Vt3.1 toxin an excellent tool for the study of neuronal BK channels where the β4 subunit is highly expressed (Li et al., 2014). Li et al. (2014) have suggested that this toxin affects the gating mechanism of the channel and induces a shift in G-V relation to more positive voltages.

### Fatty acids

The well-known role of BK channels in the regulation of vascular tone and their high expression in neurons have increased current interest in evaluating the effects of cardiovascular and nervous system regulators (such as fatty acids) on the biophysical properties of BK channels. For instance, BK channel activation by epoxyeicosatrienoic acids (EET), which are arachidonic acid metabolites, has been observed to result in concentration-dependent coronary artery relaxation (Eckman et al., 1998).

**Figure 6:**
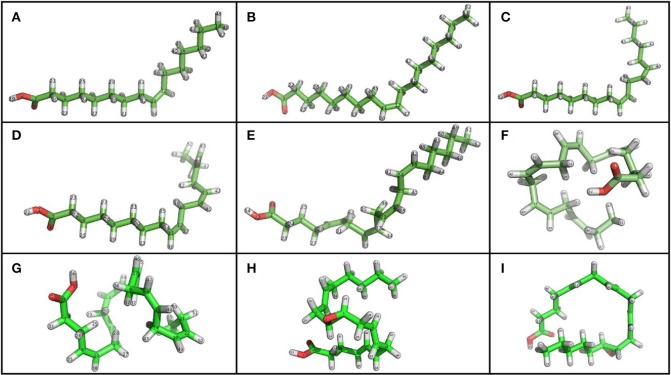
**Chemical structure of unsaturated fatty acids that modulate BK channel activity**. **(A)** PAM, Palmitoleic Acid; **(B)** OA, Oleic Acid; **(C)** LA, Linoleic Acid; **(D)** ALA, Linolenic Acid; **(E)** AA, Arachidonic Acid; **(F)** EPA, Eicosapentaenoic Acid; **(G)** DHA, Docosahexaenoic acid; **(H)** 11, 12 EET, 11, 12 Epoxyeicosatrienoic acid; **(I)** 14, 15 EET, 14, 15 Epoxyeicosatrienoic acid. Using PyMOL Molecular Graphics System, Version 1.5.0.4 Schrödinger, LL. *Green, carbon atoms; white, hydrogen atoms; red, oxygen atoms*.

Recent studies have found that unsaturated *cis* fatty acids, such as palmitoleic (PAM), oleic (OA), linoleic (LA), linolenic (ALA), arachidonic (AA), and eicosapentaenoic acids (EPA) (Figures 6A–F), significantly heighten BK channel activity by increasing their open probability (*P_0_*) without affecting channel voltage sensitivity or unitary conductance (Ahn et al., 1994; Denson et al., 2000; Clarke et al., 2002; Zheng et al., 2005). However, saturated and *trans*-unsaturated fatty acids have no effect on channel activity. In addition, it has been demonstrated that AA metabolites, such as 11,12-EET, and 14,15-EET (Figures 6G,H), increase BK channel *P_0_* in cells from both pig coronary arteries and rat pituitary GH3 with no effect on their unitary conductance and Ca^2+^ sensitivity, hence providing evidence for the direct influence of EET on the channel protein (Baron et al., 1997; Wu et al., 2000a). This was further corroborated by using inhibitors such as ChTX and TEA, which diminish or abolish the effects induced by EET on *P_0_* in bovine coronary arterial smooth muscle cells (Campbell et al., 1996). On the other hand, results observed by Fukao et al. (2001) suggest that 11, 12-EET directly acts on the α subunit independently from the β-subunit. The role of the α subunit complex as a high affinity receptor for fatty acids has been confirmed with docosahexaenoic acid (DHA), which activates BK channels from excised patches in a reversible way (Hoshi et al., 2013a). Recent studies using human BK mutants (Y318S) showed a significant decrease in the amplifying effect of DHA (Figure 6I) and EPA currents, suggesting that this residue located in the S6 segment is either part of the fatty acid binding site or constitutes an important piece of the coupling system that transforms chemical binding energy into pore-opening energy (Hoshi et al., 2013a). However, no changes were observed when AA or ALA was used (Hoshi et al., 2013c), indicating that additional α subunit residues or auxiliary subunits may be required for the enhancing effects of these fatty acids on BK channels.

In order to increase BK channel activity, studies thus far have shown that fatty acids must have the following molecular characteristics: more than 8 carbon atoms, one or more *cis* unsaturations and a negative charge (Ahn et al., 1994; Denson et al., 2000; Zheng et al., 2005). Neutral and short-chain lipids have no effect on *P_0_*, while positively charged lipids (like sphingosine) produced a decrease in N*P_0_*. Increases in *P_0_* result from decreases in mean closed time (τ_*c*_) without modifying mean open time (τ_*o*_) (Clarke et al., 2002, 2003).

Similar to the differential effects seen from toxins in the presence of auxiliary subunits, the effects of fatty acids on BK channels are modified by β subunit expression where fatty acids interacts directly with β subunits (Martín et al., 2013). When examining AA effects on BK channels formed by α + β2 or α + β3 subunits, a potentiation in the current and a slowdown in inactivation (α + β2 channels) were displayed. It is thought that this effect could be mediated by the direct interaction of AA with the channel rather than through its metabolites (Sun et al., 2007). When BK channels are modulated by AA, β subunits have important implications for the adequate function of the nervous system, considering that the expression of β2 and β3 are specific to certain types of neurons. In the absence of β2 or β3 or in the presence of β4, AA does not induce a significant response to BK channels. Furthermore, AA causes an increase in *P*_0_ as well as in the number of open channels. Effects on peak current amplitude and α + β2 current inactivation kinetics have been observed to be dose-dependent in the range of 1–50 μM of AA. At higher AA doses, a complete loss of the inactivation component was evident. Data supports the premise that the effects of AA are mediated by changes in trypsin-sensitive inactivation and that there is no obvious fatty acid-mediated activation effect upon removing inactivation. As a whole, the β2-dependent BK channel inactivation gate is a specific molecular target for AA and other unsaturated fatty acids (Sun et al., 2007). In addition, it has been shown that DHA and oleic acid (OA) can also activate α + β2 current and slow down inactivation kinetics, whereas saturated fatty acids (i.e., palmitic, stearic, and caprylic acid) seem to have no effects. Of all the fatty acids tested, OA is the most potent in enhancing BK currents.

In native human vascular smooth muscle cells, where the β1 subunit is expressed, single channel recordings have shown an important rise in the *P_0_* of BK channels induced by AA acid (Martín et al., 2013). In addition, AA accelerates voltage-dependent activation kinetics of channel. Studies in HEK 293T cells have concluded that β1 subunit expression is needed for AA to activate the channel, since no effect has been seen when the β1 subunit is not expressed. Therefore, the β1 subunit is required to mediate AA effects on BK channels by a mechanism that involves modifying gating ring operation independently from channel voltage sensitivity changes (Martín et al., 2013). In conclusion, these results strongly suggest that BK channel activation by AA requires the expression of β1, β2, or β3 subunits. Although such mechanisms have not yet been fully established, Martín et al. (2013) have suggested a mechanism for AA action that involves an increase in *P*_0_, while Sun et al. (2007) have proposed that AA prevents inactivation when BK channels are formed by the α + β2 subunit.

The degree of DHA modulation, on the other hand, depends on which β subunit is associated with the α subunit. For instance, when β1 or β4 are expressed, a large increase in peak current can be observed, but in α + β2 or αBK channels the response is rather limited. In addition, DHA differentially influences activation or deactivation kinetics, depending on which β subunit is present (β1, β2, and β4) (Hoshi et al., 2013c). It has been reported that DHA and EPA activate BK channels expressing β1 subunits (Lai et al., 2009; Hoshi et al., 2013c), and DHA has a ~20-fold current-enhancing effect on channels expressing α + β1. This effect is associated with a leftward shift in half-activation voltage, which also destabilizes the closed conformation of the conduction gate and decreases the activation time constant. The following two important residues determine the modulatory effects exerted by DHA in β1 and β4 subunits: one residue is in the N terminus (R11 in β1 and E12 in β4) and the other in TM1 (C18 in β1 and R19 in β4). The exchange of these residues with the equivalent amino acids in β2 allows this subunit (as is the case of β1 and β4) to behave as an enhancer of the DHA effect (Hoshi et al., 2013c).

In contrast to the enhancing effect found from fatty acids, a metabolic intermediary in the synthesis of fatty acids (i.e., acyl-CoA) has been shown to interact with the β2 subunit in inhibiting BK currents (Sun et al., 2008). In cerebral ischemic damage, selective AA reincorporation and accumulation into brain membranes has been observed (Rabin et al., 1997). In addition, it is acknowledged from studies in animal models that BK channel activation can potentially protect neurons under ischemic conditions (Rabin et al., 1997; Gribkoff et al., 2001). Moreover, the effects of unsaturated fatty acids on BK channel activity may influence neuronal survival/death, as their concentration may undergo rapid changes under certain pathophysiological conditions (Sun et al., 2007). Therefore, BK channel modulation by acyl-CoA or unsaturated fatty acids may control events leading to cell protection or cell death. Understanding the role of lipids under ion channel modulation conditions may lead to new therapeutic uses (Sun et al., 2008).

### Ethanol

Ethanol is a well-known modulator of the BK channel activity, as it has been described for several cells types such as neurons from rat dorsal root ganglia, nucleus accumbens and neurohypohysial nerve terminals (reviewed in Dopico et al., 1999a; Gruss et al., 2001; Knott et al., 2002; Martin et al., 2004). Changes in neuropeptide release (Knott et al., 2002) and cerebrovascular tone variations (Bukiya et al., 2009) are some of the reported effects of ethanol, all of which are reversible and seen at clinically relevant concentrations. It was initially proposed that such effects were related to the number of functional channels, changes in single channel conductance or modifications in gating properties. Subsequently, however, it was shown that gating properties were modified by the direct binding of ethanol to BK channels and by the activation of intracellular signaling cascades that may regulate the channel (Dopico et al., 1996). More recently two reports have described BK amino acid residues required for activation (Bukiya et al., 2014; Davis et al., 2014). The BK single-point mutant T352I located in RCK1 was found to be utterly insensitive to ethanol, with no changes in conductance, selectivity or gating (Davis et al., 2014). On the other hand, Bukiya et al. (2014) unveiled an alcohol-sensing site, site that is only accessible to ethanol in the presence of Ca^2+^. This site is located in the Ca^2+^-sensing tail domain and molecular modeling suggest that residue K361forms an essential hydrogen bond with ethanol only in the presence of Ca^2+^.

Studies on *mslo* (Dopico et al., 1998; reviewed in Dopico et al., 1999a), *hslo* (Feinberg-Zadek and Treistman, 2007; Feinberg-Zadek et al., 2008) and *bslo* channels (Dopico, 2003) have shown a concentration-dependent increase in *NP_0_* related to gating channel properties and not to changes in ionic conductance (Dopico et al., 1996, 1998). BK channel response is known to be voltage-independent, but is inextricably affected by intracellular Ca^2+^ concentration changes. For instance, the potentiation of *mslo* activity induced by ethanol decreases when intracellular Ca^2+^ increases, meaning that BK channel activation by this alcohol is more marked when intracellular Ca^2+^ is near basal conditions (Dopico et al., 1996, 1999a). Effects on *NP*_0_ are induced by a combination of increases in τ_*0*_ and decreases in τ_*c*_ (Dopico et al., 1998).

It has also been observed that ethanol can increase BK channel activity in a tissue-dependent manner (Dopico et al., 1996, 1999b; Gruss et al., 2001; Knott et al., 2002), which was initially related to BK isoforms and/or differential β subunit expression in various tissues (Dopico, 2003). Of the cloned β subunits, only β1, and β4 were studied and showed different responses. In vascular smooth muscles, where the β1 subunit is predominant (reviewed in Orio et al., 2002), ethanol effects depend on intracellular Ca^2+^ concentrations (Bukiya et al., 2009). For instance, when a BK channel-forming α-subunit isoform cloned from rat cerebral artery myocytes (cbv1) is expressed in *Xenopus laevis*, current potentiation after ethanol exposure (as seen by decreases in *V*_1/2_) is evident at Ca^2+^ concentrations less than 20 μM. On the other hand, rises in Ca^2+^ concentrations diminish potentiation and concentrations' over 30 μM result in inhibition. The latter response denotes that basal Ca^2+^ concentrations promote BK channel potentiation, whereas high concentrations (>10 μM) induce inhibition. Ethanol does not generate changes in channel unitary current amplitude, which implies that its effect on cbv1 channels is limited to modifying gating. Finally, the effect of ethanol depends on the intracellular Ca^2+^ levels sensed by the α subunit (Bukiya et al., 2009).

In studies, where cbv1 was co-expressed with the β1 subunit, shifts from ethanol induced activation to inhibition were found at Ca^2+^ concentrations less than 3 μM. The latter implies that β1 subunit expression induces BK channel current inhibition by ethanol at intracellular Ca^2+^ levels similar to those reached during cerebral myocyte contraction (4–30 μM). These results indicate that β1 subunit effect on ethanol activity is mediated with an allosteric mechanism where β1 acts like a transducer that couples changes in Ca^2+^ concentration to BK channel activation (Bukiya et al., 2009). Similar results were also found in myocytes, indicating that the β1 subunit would be responsible for ethanol-induced BK current inhibition at membrane potentials and Ca^2+^ concentrations reached during cell contraction (Bukiya et al., 2009).

In experiments performed on soma and dendrites of nucleus accumbens neurons, BK channels exhibit a dual behavior, where somatic channels are sensitive to ethanol, while dendritic channels are not. In other words, when the β4 subunit is expressed, ethanol has a potentiating effect, whereas when β1 is expressed, this effect is not exerted. These results were further confirmed by heterologous expressions of α and β4 subunits in HEK293 cells (Martin et al., 2004; Treistman and Martin, 2009). The increase of the α + β4 channel's *NP_0_* results from a rise in τ_*o*_ and a fall in τ_*c*_ (Feinberg-Zadek and Treistman, 2007).

The effect of β1 and β4 in the response of BK channels to ethanol has been related to alcohol tolerance, implying a loss of effectiveness with time, which is a key feature of addiction (Martin et al., 2008). The mechanism by which β subunits determine BK channel potentiation seems to also modulate alcohol sensitivity, which may contribute to explaining the roles of genetic predisposition and tissue-dependent expression of BK channels (Feinberg-Zadek and Treistman, 2007). For example, β1 determines low BK channel sensitivity to ethanol (Feinberg-Zadek et al., 2008), whereas β4 subunit expression in BK channels potentiates the response to ethanol. This suggests that in the absence of the β4 subunit, acute tolerance of BK channels is induced, while the presence of this subunit abolishes it. Accordingly, there is a strong association between alcohol tolerance and predisposition to alcoholism. Therefore, the β4 subunit could determine specific differences related to alcohol abuse and alcoholism, hence making it a potential therapeutic target (Martin et al., 2008). Intermittent chronic alcohol exposure could increase expression of β1 subunits in local brain regions. The latter could be used as a good strategy in alcohol-dependent subjects to selectively alter their motivational drive to excessively consume alcohol (Kreifeldt et al., 2013).

### Steroids

Steroids usually exert their action through specific receptors within cells. The lipophilic nature of these components benefits their entrance across the cellular membrane to the cytoplasm, allowing them to bind to receptors acting as transcription factors in the nucleus. (Thiede et al., 2012). Nevertheless, there are certain rapid effects that are independent of this nuclear signalization and start in membrane receptors (Simoncini et al., 2004).

**Figure 7:**
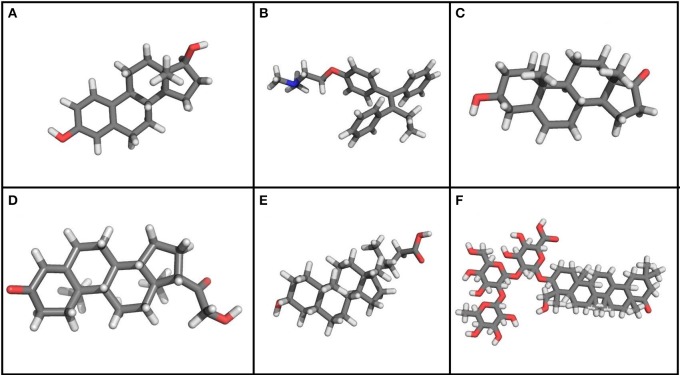
**Chemical structure of steroids and other molecules that modulate BK channel activity**. **(A)** 17β-Estradiol. **(B)** Tamoxifen. **(C)** Dehydroepiandrosterone. **(D)** Corticoesterone. **(E)** Lithocholic acid. **(F)** Dehydrosoyasaponin-I. Using PyMOL Molecular Graphics System, Version 1.5.0.4 Schrödinger, LL. *Gray, carbon atoms; white, hydrogen atoms; red, oxygen atoms; blue, nitrogen atoms*.

17β-estradiol (E2) (Figure 7A) has been shown to activate BK channels in a nuclear receptor-independent manner. This activation is caracterized by an increase in potassium currents, accelerated current kinetics and a rise in *P_0_* to less positive potentials at 1–20 μM of E2 and at low Ca^+2^ concentrations (~100 nM) (Valverde et al., 1999; De Wet et al., 2006). The observed increments in BK activity are related to the expression of auxiliary β subunits, that can act like a receptor for E2 (King et al., 2006). Such increments can be β1-dependent at <1 μM of E2 or β1-, β2-, and β4- dependent at 1–30 μM, (Behrens et al., 2000; King et al., 2006), which suggests the existence of an E2 membrane receptor that can distinguish between different β subunits. (King et al., 2006). The action of E2 on BK channels is determined by type of β subunit expression, and it can be observed in native (Holdiman et al., 2002; Coiret et al., 2005; Ohya et al., 2005), and heterologous expression (Valverde et al., 1999; De Wet et al., 2006).

Although E2 can activate the BK channel when the β1 subunit is present, the exact binding site has not yet been identified. E2 activation can be induced when E2 is conjugated to a protein that is impermeable to the membrane, but only when it is applied to the outside of the cell, suggesting that the binding site is located in the extracellular region of the β1 subunit (Valverde et al., 1999). Regulation in proteasomal degradation of the β1 subunit in the presence of E2 also suggests the importance of this subunit for cellular metabolism (Korovkina et al., 2004; Nagar et al., 2005).

After identifying the stimulating activity of E2 on BK channel properties, research has focused on other molecules that can exert the same characteristics. It has been shown that Tamoxifen (Figure 7B), which is an agonist of estrogen receptors that is widely used for breast cancer therapy, also activates the BK channel, but is at least 10 times more potent than E2, causing a reversible and fast effect at concentrations as low as 0.1 μM (Dick et al., 2001). The E2-mediated increase in *P*_0_ was also observed in the absence of cytoplasmic signals, as this effect was observed in BK channels incorporated into lipid bilayers, as well as in outside out and inside out patches. Changes in channel activation, however, were not seen when the BK α subunit was alone, as confirmed in experiments with β1—knockout mice cells (Dick and Sanders, 2001). Ethylbromide tamoxifen, which is a molecule that does not permeate the membrane, was observed to elevate potassium current at +40 mV and also raise the open probability when applied to the outside of the membrane, indicating that there may be an extracellular binding site (Dick et al., 2002) just as was proposed for E2 (Valverde et al., 1999).

Dehydroepiandrosterone (DHEA; Figure 7C), which is an adrenal androgen, is another steroid derivative that activates BK channels, for which such sensitivity is provided by the β2 subunit. Corticoesterone (Figure 7D) at physiological concentrations can also quickly and reversibly activate BK channels when expressed with the β4 subunit. Under conditions of constant calcium concentration, it primarily acts by shifting the voltage activation curve to the left. This finding has suggested that β subunits differentiate between steroids, but that BK channels do not respond when α subunits are present alone (King et al., 2006), which has led to an increased interest in researching the molecules targeting the β1 subunit as a potential modulator of the BK channel. Lithocholic acid (Figure 7E), being a cholane at micromolar concentrations (EC_50_ = 45 μM), acts as a BK channel activator only in channels formed by α + β1 (Bukiya et al., 2013).

The binding site of these molecules is another important research focus, due to its pharmacological potential. Structural molecular models and structure–activity relationship (SAR) studies have led to the identification of a binding site for lithocolic acid in the β1 subunit TM2 domain (Bukiya et al., 2011). The search for similarities in such models and structural databases is helpful for the identification of other molecules, such as sodium 3-hydroxyolean-12-en-30-oate (HENA). This compound is not a steroid, but it activates BK channels because it contains the structural characteristics considered to be necessary for its activation and, interestingly, it also activates BK channels only when β1 subunits are expressed (Bukiya et al., 2013).

Dehydrosoyasaponin-I (DHS-I) (Figure 7F) isolated from *Desmodium adscendens* is a reversible and potent activator of the BK channel. When applied to the intracellular side of the membrane, it causes a shift in both calcium- and voltage-dependent activation curves. It was identified because it inhibits ChTX biding to the channel in bovine tracheal smooth muscle membranes (McManus et al., 1993). A triterpene glycoside was the first and most potent activator to be identified at nanomolecular (100 nM) concentrations, causing an 80 mV lefward shift in *V*_1/2_ and an increase in open probability by means of a direct mechanism (Giangiacomo et al., 1998). The identification of this effect in smooth muscle membranes has led to report that such activation depends on the expression of the β1 subunit, although it is unclear whether this effect is determined by other subunits as well (Bukiya et al., 2012).

### Other molecules

Tungstate is a molecule commonly used as and antihypertensive agent (Swei et al., 1999) that modulates BK channels expressing α subunits alone or coexpressed with β subunits (β1 – β4) in a concentration-dependent manner (Fernández-Mariño et al., 2012). When comparing 0.1 and 1 mM of tungstate, it was observed that both concentrations can induce a decrease in V_1/2_, but reductions in current amplitude are seen only at higher concentrations. V_1/2_ declines are calcium-dependent and are only seen in the presence of β1 or β4 subunits, but not in channels containing β2 or β3 subunits (Fernández-Mariño et al., 2012). The tungstate putative binding site is located in the BK channel α subunit, but residues from the β1 extracellular loop are also required for the appropriate activation of the channel. It has been proposed that extracellular loop residues are involved in maintaining the BK channel structure needed for tungstate to bind to the channel (Fernández-Mariño et al., 2014).

Another BK channel activator is DiBAC_4_ (bis (1,3)-dibutylbarbituric acid) trimethine oxonol), which is a voltage-sensitive fluorescent dye that significantly increases whole-cell BK α + β1 currents in HEK293 cells and in native channels from urinary bladder smooth muscle cells. DiBAC_4_ shifts the activation voltage to hyperpolarizing potentials without causing changes in single-channel conductance. Morimoto et al. (2007) reported that DiBAC_4_ activates whole-cell rBKα + β1 and rBKα + β4 currents partially blocking rBKα + β2 currents with no effects on channels where the α subunit is alone (Morimoto et al., 2007). Nonetheless, Scornik et al. (2013) suggested that the DiBAC_4_ binding site should be in the α subunit, since DiBAC_4_ selectively enhances BK channel activity in the presence or absence of the β1 subunit (Scornik et al., 2013). It has been shown that DiBAC_4_ can increase deactivation time constants without changing the voltage dependence of deactivation. Although the latter effects do not necessarily require the presence of β1 subunits, these subunits enhance BK channel response to DiBAC_4_. It has also been established that leftward shifts in conductance-voltage curves induced by DiBAC_4_ are significantly larger in channels expressing α + β1 than in those expressing α subunits alone. Moreover, β1 subunits promote a four-fold decrease in the *K_d_* of DiBAC_4_. Such findings could envision a new scope for the role β1 subunits in cardiovascular pharmacology (Bosch et al., 2014).

Additionally, NO, being an endogenous vasodilatation gas, can rapidly, reversibly, and significantly increase BK open channel probability from both sides of the membrane (Bolotina et al., 1994; Peng et al., 1996). Carbon monoxide (CO), which is another endogenous like NO, can increase native BK channel activity. However, stimulatory effects of CO and NO rely on specific interactions, where CO interacts with α and NO with β subunits (Wu et al., 2002). The alkaloid tetrandine is a blocker of *hslo* current and its action as a BK inhibitor is potentiated in channels coexpressing α and β subunits (Dworetzky et al., 1996). In endothelial human cells, it was found that tetrandrine shifted the G-V to more positive potentials and inhibited the maximal *P*_0_ (Wu et al., 2000b). BK channel expression in human gliomas appears to be correlated with tumor malignancy grade and is involved in the proliferation of human osteoblasts (Henney et al., 2009; Chen and Tseng, 2010).

## Concluding remarks

BK channels are related to different pathophysiological processes, such as changes in vascular tone regulation, diabetes, kidney, and nervous system diseases. The expression of auxiliary β subunits plays an important role in these processes because of the modulatory effects of these subunits on BK channel activity, inducing changes in their biophysical properties. Furthermore, β subunits act like binding sites for various molecules that regulate BK channel activity, prompting an increase or decrease in the effects induced by drugs. This knowledge would be important in drug design, since it may be possible to find molecules that induce specific modulatory effects on BK channel properties, depending on the tissue types where they are expressed. That is particularly important in smooth muscles and in the nervous system, where β1 and β4 are highly expressed and have been found to significantly regulate the biophysical properties of BK channels.

### Conflict of interest statement

The authors declare that the research was conducted in the absence of any commercial or financial relationships that could be construed as a potential conflict of interest.
